# Sampling event dataset for ecological monitoring of riparian restoration effort in Colorado foothills

**DOI:** 10.3897/BDJ.8.e51817

**Published:** 2020-04-03

**Authors:** Richard Levy, Margo Paces, Rebecca Hufft

**Affiliations:** 1 Denver Botanic Gardens, Denver, United States of America Denver Botanic Gardens Denver United States of America

**Keywords:** sampling event, riparian, ecological restoration, ecological monitoring, ground cover

## Abstract

**Background:**

The foothills and shortgrass prairie ecosystems of Colorado, United States, have undergone substantial and sustained anthropogenic habitat change over the past two centuries. Riparian systems have been dramatically altered by agriculture, hydrological engineering, urbanisation and the introduction of non-native invasive species. In 2016, Denver Botanic Gardens began a restoration effort of Deer Creek which seeks to modify the hydrology of the creek by mimicking the effects of beaver dams with artificial structures. The site, owned by the US Army Core of Engineers and managed by Denver Botanic Gardens, had been the subject of previous botanical surveys. With the initiation of the restoration project, permanent transects were established along the stream and are sampled for ground vegetation richness and abundance, canopy cover, soil and stream conditions and aquatic macroinvertebrate community makeup on an annual basis. To provide a means for tracking any post-intervention changes in the riparian ecosystem, this resource reports all recorded occurrences and measurements, along with methodologies and motivations from past and current surveys in the form of a sampling event dataset.

**New information:**

The current project and past surveys document 382 plant taxa and 157 aquatic macroinvertebrate taxa. A total of 16304 occurrences and 7422 measurements are included in the resource. Occurrence and measurement data taken from transects provide a means to measure species abundance, ground cover and other biotic and abiotic characteristics relevant to assessing the effects of hydrological restoration on riparian plant communities.

## Introduction

Riparian corridors and waterways in the American West have been drastically altered though agricultural disturbance, hydrological engineering, exotic species introductions, urbanisation and historical exploitation of the North American beaver, *Castor canadensis* (Poff et al. 2011, Naiman et al. 1988). Within this arid region, amid a complex matrix of fragmented habitat and land use change, waterways are crucial resources for ecosystem services and biodiversity refugia (Palmer et al. 2013, Seavy et al. 2009, Sweeney et al. 2004).

In 2016, Denver Botanic Gardens initiated long-term ecological restoration of Deer Creek in southern Jefferson County, Colorado, United States. A section of Deer Creek runs from west to east through a Jefferson County Open Space property and Denver Botanic Gardens Chatfield Farms property. The creek and its surrounding area had been subjected to human disturbance such as channelling, livestock grazing, hay production and urbanisation, dating back as far as the mid-19^th^ century (Colorado Encyclopedia 2016). Such disturbances have contributed to an overwhelming dominance of non-native plant species in the understorey of the riparian area along Deer Creek, as well as the presence of non-native tree species within the overstorey. The hydrology of the creek was modified, resulting in increased flow energy, run-off volume and intensity and deepened, undercut channels. The restoration project saw the installation of small channel structures that function like beaver dams to facilitate over-bank flows to move water from the stream channel and distribute it across the floodplain. We hypothesise that these techniques will restore the hydrological conditions suitable for the regeneration of native riparian plant species through active and passive measures.

To track progress, we established permanent transects to monitor the ground vegetation community, canopy cover, stream conditions, water quality and aquatic macroinvertebrate diversity. Presented as a sampling event dataset, using the Darwin Core standard and relevant extensions, we provide records for all data, samples and specimens pertaining to the restoration project’s progress, as well as specimen data from previous surveys in the area of interest (Levy et al. 2020).

## General description

### Purpose

Sampling transect data were recorded to monitor any changes in ground vegetation, canopy cover, water quality and aquatic invertebrate community over the course of an ongoing restoration effort of the hydrological conditions of the stream. Herbarium specimens were collected in previous and the current surveys to document plant species richness in the areas described in this dataset and are provided here for additional context.

## Project description

### Title

Deer Creek Riparian Restoration Ecological Monitoring

### Personnel

- Rebecca Hufft. Associate Director of Applied Conservation. Denver Botanic Gardens. https://orcid.org/0000-0002-8404-2712
- Margo Paces. Graduate Student and Botany Seasonal. University of Colorado, Denver. Denver Botanic Gardens. https://orcid.org/0000-0002-0221-4921
- Richard Levy. Database Associate. Denver Botanic Gardens. https://orcid.org/0000-0002-4401-1380

### Study area description

The study area lies within the Deer Creek Sub Watershed (Hydrological Unit Code: HUC 12 101900020702) which lies within the Upper South Platte River Watershed (HUC 8 10190002). This area, known as the Chatfield Basin, is located at the base of the foothills of the Eastern Slope of the Rockies at the intersection of the Southern Rockies, High Plains and South-western Tablelands Level III Ecoregions (Chapman et al. 2006). Deer Creek is a stream that flows from west to east, in southern Jefferson County, Colorado. The 4.7 km section of stream (and adjacent area) that is being monitored is located just west of Chatfield Reservoir. Monitoring sites are located within the Hildebrand Ranch Park (Jefferson County Open Space) and Denver Botanic Gardens Chatfield Farms. Monitoring sites were selected to represent sections of stream upstream, within and downstream of hydrological manipulation sites, within the extents of the properties granting permission for the survey that had sufficient bank vegetation accessible for a 25 m transect parallel to the creek. Hydrological manipulation, with the aim of restoring natural stream conditions, is being conducted only within the Denver Botanic Gardens Chatifeld Farms property, at three distinct sites within the streambed (locationID = Deercreek04, DeerCreek05, DeerCreek06), where historical oxbows were thought to exist. In total, 18 ecological monitoring sites have been established along this section of the creek. The habitat is characterised by historical ranching, previous and ongoing agricultural practices, hydrological manipulation, an understorey dominated by non-native plant species and a tree canopy consisting primarily of cottonwood (*Populus*) species in the riparian zone. During the first four years of ecological monitoring (2016-2019), the region's temperate climate experienced an average of 529 mm of precipitation and a mean temperature of 10.25°C , with a mean minimum and maximum temperature of 2.275 and 18.3°C , respectively (PRISM Climate Group, Oregon State Univeristy 2019).

### Design description

**Figure 1.**
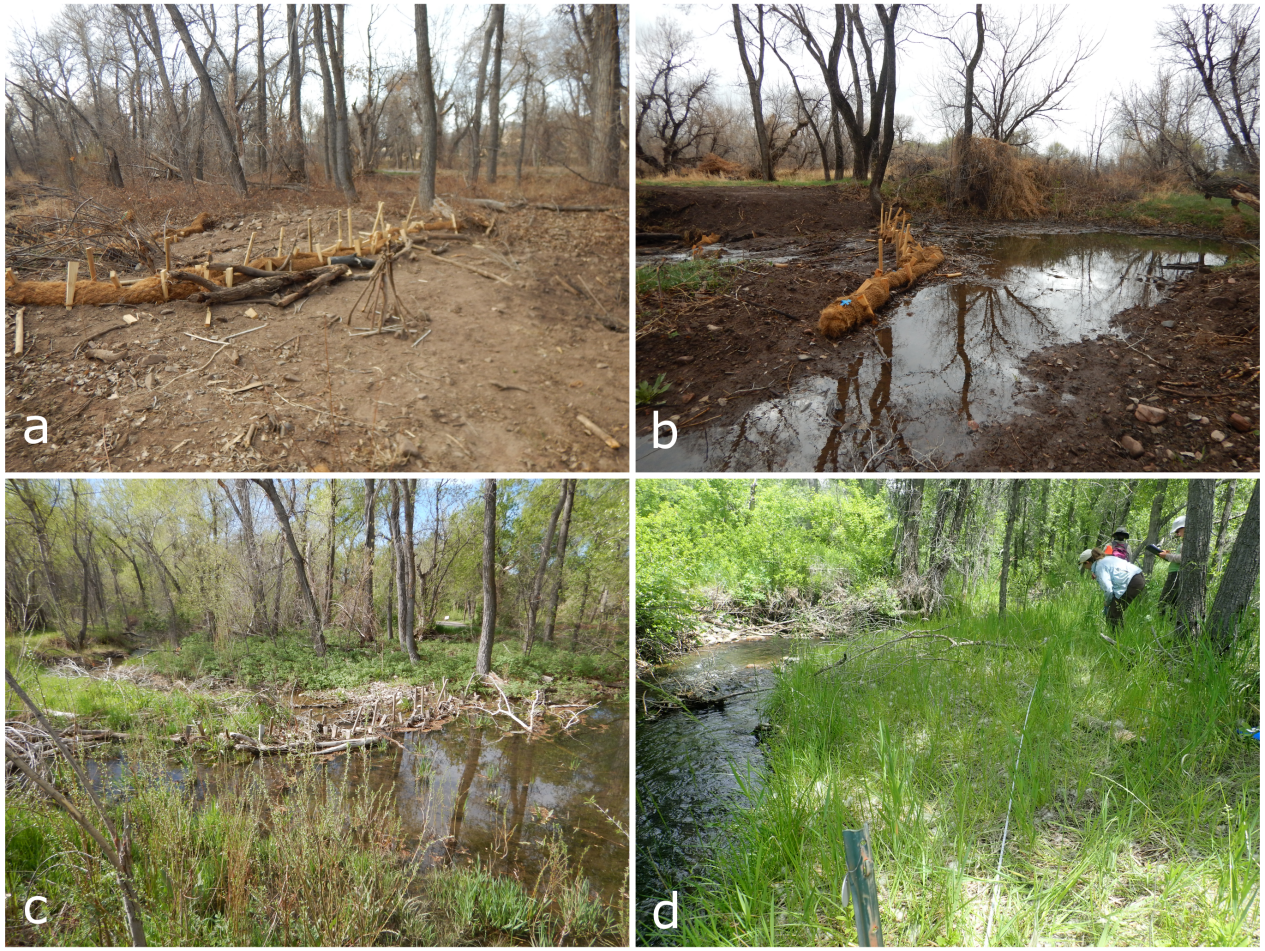
Images of artifical beaver dam channel structures and vegetation monitoring transect. **a)** Artificial beaver dam structure at locationID DeerCreek05 on 17 March 2017 with dry creekbed. **b)** Artificial beaver dam structure at locationID DeerCreek05 on 29 March 2017 with wetted creekbed. **c)** Artificial beaver dam structure at locationID DeerCreek05 on 10 May 2018 with wetted creekbed during growing season. **d)** Vegetation monitoring transect at locationID DeerCreek18 on 26 June 2019.

Temporary structures, designed to mimic beaver dams (Fig. 1), were installed in three locations within the Deer Creek stream bed (locationID = DeerCreek04, DeerCreek05, DeerCreek06) to facilitate over-bank flows and wetting of larger floodplain areas and to restore hydrological conditions suitable for the regeneration of cottonwoods and willows. While beavers are present in the immediate and surrounding areas, artificial dam structures were installed to accelerate the recovery of the stream, doing so in locations which are accessible, beneficial for educational outreach efforts and where historical oxbows are thought to have existed and will not impact existing infrastructure. Monitoring of these three locations and fifteen additional downstream and upstream sites was designed to document and describe the ground vegetation community, soil moisture conditions and canopy cover. Belt transects at each site were 25 m long and sampled via the point-intercept method (Hufft et al. 2019a adapted from Herrick et al. 2005). Reaches of the stream, adjacent to the transects, were sampled for water quality and macroinvertebrate community diversity.

### Funding

Funding was provided by the Borgen Family Foundation, National Fish and Wildlife Foundation Five Star and Urban Waters Program, Colorado Department of Agriculture Noxious Weed Fund, Denver Debutante Ball, Jefferson County Open Space and Colorado Water Conservation Board.

## Sampling methods

### Study extent

Data were collected in 2015 through 2019. In 2015, a botanical survey was conducted to record and voucher plant species occurring in the area of interest for restoration. Twelve monitoring sites were established in 2016 and six were added in 2018 for a total of eighteen sites. Each site has a permanent 25-m belt transect that was surveyed once per year during the summer months. Stream and water conditions in points of stream, adjacent to transects, were sampled once per year, as were aquatic macroinvertebrates. Specimens housed in the Kathryn Kalmbach Herbarium (KHD), collected during previous surveys (Alba and Islam 2019, GBIF.org 2019) of the area, are also included in the data resource. These surveys were conducted as inventories to document species richness of the area.

### Sampling description

All sites were sampled once per year, during the growing season when most vegetation was mature enough to make proper species identifications.

For the first two years of monitoring (2016-2017), twelve 25-m transects were installed to measure ground and canopy cover. For ground cover, every 0.25 m was sampled via the point intercept method. Ground vegetation species were recorded as top canopy (1st hit) or lower canopy (2nd hits) and presented here as occurrences, based on human observation. In 2016 and 2017, the ground surface type was recorded only when no vegetation was present, but beginning in 2018, ground surface type was recorded every 0.25 m. Ground surface type is provided in the extendedMeasurementOrFact extension within this resource. When no vegetation was present at a point along the transect, we provide this information in the form of an occurrence record with the status "absent" and indicate the type of ground cover in the occurrenceRemarks. For canopy cover, every 0.5 m was sampled. Canopy species observed through a GRS densiometer were recorded and are provided here as occurrences, based on human observation. Any plant species observed within 1 m of either side of the transect, that were not recorded as part of the point intercept sampling, were also recorded as observation occurrences. Voucher specimens of plants were taken when identification to species was not confident or possible in the field. Sections of stream, directly adjacent to the vegetation transects, were sampled for stream conditions and water quality measurements, including stream velocity, stream depth, stream width, bank height, bank to thalweg distance, streambed substrate at thalweg, water surface to bankful distance, wetted channel width, estimated percent of pools, estimated percent of runs, estimated percent of riffles, estimated percent of undercut bank, water nitrogen levels, *E. coli* content, dissolved oxygen, water temperature, electrical conductivity, pH, coliform levels, total dissolved solids and visual condition of water in stream. Water samples were sent to a contractor for measurements. Aquatic macroinvertebrate communities were also sampled within the stream and sent to a contractor for sorting and identification.

In 2018, six additional transects were added upstream of the temporarily installed dam-like structures. Several additional measurements were also recorded along the vegetation transects: soil moisture percentage was measured every 1.0 m, canopy cover percentage was measured with a spherical densiometer every 0.5 m and ground surface type, regardless of the presence of vegetation, was recorded every 0.25 m. These measurements are provided within the extendedMesurementOrFact extension of this resource.

Voucher specimens from previous botanic surveys, aimed at documenting species richness of the area, are also included in this resource as occurrences, based on preserved specimens. The property on the eastern portion of the stream reach is managed by Denver Botanic Gardens and, consequently, has been an area of thorough botanical survey. Specimen occurrence data from these varied botanical surveys is provided here to document the historical existence and/or location of plant species in the area, as this passive long-term restoration effort may rely on seedbanks and the movement or expansion of existing local populations. Methods used in previous surveys were not as well documented, but are similiar to those in Alba and Islam (2019) with the purpose of producing species checklists. Specimen occurrence data were downloaded from the Kathryn Kalmbach Herbarium Occurrence Dataset via the Global Biodiversity Information Facility (Kathryn Kalmbach Herbarium (Denver Botanic Gardens) 2019, GBIF.org 2019) and subsequently limited to those falling within the immediate vicinity of the riparian zone of Deer Creek (within 0.0005 degrees or 116 m of Deer Creek) (U.S. Geological Survey 2018).

### Quality control

Raw data from sampling transects were checked upon transcription into digital format. Once data were submitted into a relational database, a random sample of records was produced and checked against the raw data. During ecological surveys, herbarium voucher specimens were collected when species identification could not be confidently determined in the field.

### Step description

**Measuring ground cover/community composition** (Hufft et al. 2019a)

1. Pull out the 25 m tape in between two rebar posts. The line should be taut and as close to the ground as possible.
2. Take photograph at origin of transect. Stand behind the post, face the endpoint and take the picture with the post in the photo.
3. Begin at the “0” end of the line, move at 0.25 m intervals towards the end.
4. At origin (0 m), midpoint (12.5 m) and end (25 m), measure distance from transect to bank and bank height.
5. The starting point will be 0.25 m. Always stand on the same side (away from the stream) of the line.
6. Drop a pin flag to the ground from a standard height of 1 m next to the stream side of the tape. The pin should be vertical. The pin should be dropped from the same height every time. Do not guide the pin to the ground, let it fall freely.
7. Once the pin flag is on the ground, record every species it intercepts. The first species it hits (the highest one/furthest from the ground) is the “Top canopy”. If no leaf, stem or plant base is intercepted, record “NONE” in the “Top canopy” column. Record all additional species intercepted by the pin in the “Lower Canopy Layers” column. Record them in order from closest to the top canopy to furthest (highest to lowest). Record each species only once, even if it is intercepted multiple times. If species cannot be identified at the current stage, flag the plant and record location on “Unidentified Species” datasheet and “Unidentified Species” section of the vegetation datasheet. Return later to identify or collect voucher specimen of same species from outside the transect.
8. Record the ground surface the pin flag rests on. Options are litter, bare soil, rock (> 5 mm diameter), standing dead vegetation, water, downed woody debris (logs/large branches), road/trail (paved or gravel trail).

**Measuring soil moisture** (Hufft et al. 2019a)

1. Beginning at the “0” end of the transect, measure the soil moisture on the right side of the tape at 1 m intervals, starting at 1 m.
2. At each point, insert the soil moisture meter at the right side of the transect 20 cm deep into the soil.
3. Record the percent volumetric soil moisture that appears on the screen. If the soil is too hard or rocky to fully insert the moisture meter, record that the measurement was unable to be taken.

**Measuring tree canopy cover** (Hufft et al. 2019a)

1. Beginning at the “0” end of the transect and starting at the 0.5 m mark, measure the tree canopy cover every 0.5 m. Stand on the side of the tape furthest from the creek facing the end point of the transect.
2. At each point, open the spherical densiometer.
3. Hold the densiometer out so that the bubble in the corner is in the centre of its circle, indicating that the instrument is level.
4. Hold the densiometer about 30-46 cm away from you and low enough so you can see all 24 squares in the window.
5. Imagine 4 dots at each corner of each of the 24 squares. Count the number of dots in which a tree is visible.
6. Additionally, record species of every tree that appears in the densiometer window.

**Recording additional species** (Hufft et al. 2019a)

1. After measuring for percent cover, use the metre stick as a guide to search the belt transect area for any species that were not recorded while measuring percent cover.
2. Record additional species identified that are rooted within the 25 m × 2 m belt. Collect a voucher specimen, if unable to identify in the field.

**Water Quality Measurements** (Hufft et al. 2019b)

1. Start at thalweg (deepest point) adjacent to transect origin.
2. Collect three water samples in official Colorado Department of Public Health and Environment bottles:
  - Nitrate/Nitrite (250 ml)
  - Total Nitrogen (125 ml)
  - E. coli (125 ml)
3. Fill each bottle to the max fill line.
4. Attach supplied CDPHE labels.
5. Fill out CDPHE Request for Analytical Services form. Each transect needs its own testing form.
6. Each water quality test will have its own specific bottle (3 per transect for E. coli, nitrate/nitrite and total nitrogen)
  - Nutrient bottles contain an acid for preservation, so do not dump or spill bottles.
7. Take water samples at the thalweg origin or, if stream is too deep for wading, at the deepest point possible. Make sure to collect probe data and water samples at the same place and record this location on the datasheet.
8. Samples are TIME SENSITIVE. Samples must be dropped off within 8 hours of sampling. No sample drop-offs on Fridays.

**Stream Measurements** (Hufft et al. 2019b)

1. Start at thalweg adjacent to vegetation transect origin. If thalweg is too deep to wade, take measurement at the deepest point possible and record this point on datasheet.
2. For each probe, make sure to not submerge probes past the point where the storage caps seal and swish the probe in water to remove air bubbles and allow reading to stabilise before recording.
3. Using the appropriate probes, collect the following data and record on data sheet:
  - Temperature (°C)- Use pH probe’s thermometer.
  - pH
    - Probe should not be allowed to dry out. Store in pH 4 standard (or storage solution, if available).
    - If probe does dry out, it must be soaked in standard or storage solution for 1 hour before use.
  - Total Dissolved Solids (TDS)
  - Dissolved Oxygen (DO, mg/l)
    - If the probe has not been used in 7 days, it requires 3 minutes to polarise. Turn probe on and wait 3 minutes before testing.
    - Sponge in storage cap must always be moistened (but not soaked) with DI or RO water.
  - Electrical Conductivity (EC)
  - Flow
    - Beginning at thalweg at origin, find a section of reach with uniform bottom and flow.
    - If thalweg is too deep to wade, take measurement at the deepest point possible at the origin and record location on data sheet.
    - Plug cord into back of white unit, zero should appear on screen.
    - To zero counter, switch position up.
    - To pause measurements, put switch in middle.
    - To start measuring, put switch down.
    - Use timer to measure flow for 60 seconds at 60% of stream depth.
    - Once back in the office, to calculate surface velocity (m/s), convert the flowmeter readout using the following equation: V(c/m)=(0.000854C)+0.05
  - Rinse all probes with Reverse Osmosis water after use and before putting on cap to avoid contamination.

**Bank Measurements** (Hufft et al. 2019b)

1. Total Bank Height: Distance from streambed at edge of bank to top of bank.
2. Surface to bank height: Distance from the water surface level to top of bank.
3. Distance to bank from Origin: Distance from the origin rebar to the edge of the adjacent stream bank.
4. If water is too deep at the thalweg to wade in, take measurements at the deepest point possible adjacent to the origin and record location on datasheet. This is especially important for stream reaches with dams.

**Aquatic Macroinvertebrate Collection** (Hufft et al. 2019b)

1. Since the water is moving when we sample, we use the kicknetting method.
2. Start at thalweg at 20 m mark downstream from origin. Start downstream and move upstream towards origin (See Fig. 1)
3. Conduct kicknetting for 1 minute every 5 m, for five sampling bouts moving upstream.
4. Five sampling bouts should be conducted for each transect.
  - However, samples should be 5 m apart, so if large parts of the stream are dry, only take as many samples as the stream allows.
5. At each sampling bout,
  - Disturb area 1 m2 upstream of net, using heel or toe to dislodge the upper layer of cobble/gravel and scrape underlying bed.
  - Pick up larger substrate and rub by hand to remove attached organisms.
  - If the water is slow moving, use your hands or feet to push what has been kicked up into the net.
  - Exclude specimens clinging to the outside of nets.
6. Place contents of net into the mesh bucket.
  - Rinse outside of net to move sediment and specimens inside the net to one corner.
  - Flip net inside out in bucket and rinse down outside of net with more water to wash all contents into bucket.
  - To avoid contamination of the sample, do not pour water into the side of the net with the specimens. Only pour water on the outside of the net.
  - Continue adding contents of net into mesh bucket for the length of the transect.
7. Scoop specimens into 1 litre plastic rectangular Nalgene sampling jar by hand. It may be necessary to place the mesh bucket in the stream and swirl to get specimens to one side.
  - Release any fish, amphibians, reptiles or crayfish back into stream, but record their presence on the data sheet.
8. Fill jar with no more than 50% of sample material from the stream. Use more than one jar per sample, if necessary.
  - Add ethanol to the bottles to create a 50:50 sample to ethanol ratio.
  - In 2018, we used an average of 2-3 bottles per sample site (max 5 at TSP and dammed sites) and roughly 2.5 x 750 ml of ethanol per day.
9. Pick out and scrape off larger rocks in the bucket to remove any macroinvertebrates clinging to them. Smaller gravel can be added to bottle.
10. Properly label each bottle using a marker that is not alcohol-soluble. Check naming conventions file to make sure samples are labelled consistently from year to year.
11. Follow the instructions below for proper storage and sample drop-off for identification.
12. Backwash the net with stream water before collecting samples from the next site.

## Geographic coverage

### Description

**Figure 2.**
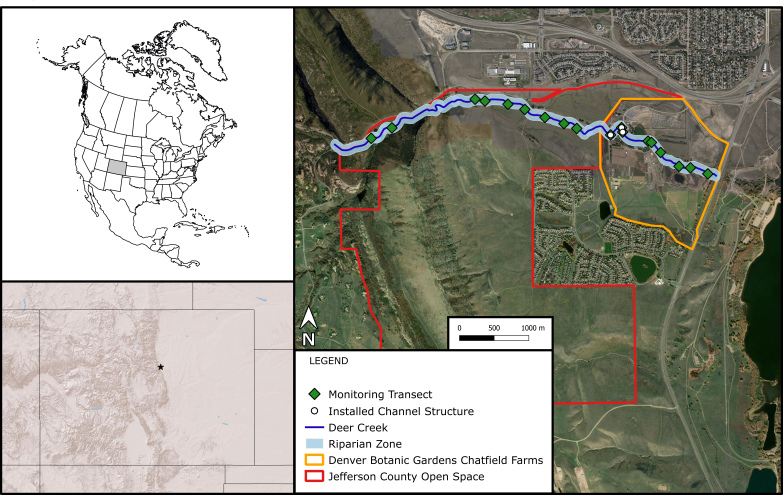
Section of Deer Creek that was monitored, including transect and channel structure locations in Colorado, United States.

Data were collected from 18 sites along Deer Creek (Fig. 2), a stream that flows from west to east through urbanised montane foothills ecosystems in Jefferson County, Colorado, United States. The 4.67-km section of stream studied here occurs on the east slope of the Front Range mountain range, where is flows through Hildebrand Ranch Park (Jefferson County Open Space) and Denver Botanic Gardens Chatfield Farms, sites of historical ranching and historical and active agriculture.

### Coordinates

39.544 and 39.555 Latitude; -105.0885 and -105.136 Longitude.

## Taxonomic coverage

### Description

Angiosperms and gymnosperms are provided as occurrences, both as preserved specimens and observations. These originate from botanical surveys, ground vegetation and canopy monitoring efforts. Invertebrate animals are provided as observation-based occurrence data. Invertebrate samples were collected as part of the aquatic macroinvertebrate surveys within the stream and sent to a contractor laboratory for identification and analysis.

### Taxa included

**Table:** 

Rank	Scientific Name	Common Name
phylum	Tracheophyta	vascular plants
kingdom	Animalia	animals

## Temporal coverage

**Data range:** 1981-6-24 – 1984-6-24; 2014-5-08 – 2014-8-14; 2015-4-28 – 2015-11-02; 2016-6-08 – 2019-7-10.

### Notes

Three botanical survey efforts were undertaken prior to implementation of restoration effort and ecological monitoring.

## Collection data

### Collection name

Kathryn Kalmbach Herbarium

### Collection identifier

http://grscicoll.org/institutional-collection/kathryn-kalmbach-herbarium

### Parent collection identifier

http://biocol.org/urn:lsid:biocol.org:col:15415

### Specimen preservation method

Dried and pressed

## Usage rights

### Use license

Creative Commons Public Domain Waiver (CC-Zero)

## Data resources

### Data package title

Deer Creek Riparian Restoration Ecological Monitoring

### Resource link

https://www.gbif.org/dataset/f61e69d1-e79f-4ccb-bd92-56a7cefcf1e4

### Alternative identifiers

https://doi.org/10.15468/scmp5u

### Number of data sets

4

### Data set 1.

#### Data set name

event.txt

#### Data format

Tab delimited Darwin Core Archive

#### Number of columns

24

#### Character set

UTF-8

#### 

**Data set 1.** 

Column label	Column description
eventID	An identifier for the set of information associated with an Event (something that occurs at a place and time). May be a global unique identifier or an identifier specific to the dataset.
parentEventID	An identifier for the broader Event that groups this and potentially other Events. http://rs.tdwg.org/dwc/terms/parentEventIDAn identifier for the broader Event that groups this and potentially other Events.
samplingProtocol	The name of, reference to, or description of the method or protocol used during an Event.
sampleSizeValue	A numeric value for a measurement of the size (time duration, length, area or volume) of a sample in a sampling event.
sampleSizeUnit	The unit of measurement of the size (time duration, length, area or volume) of a sample in a sampling event.
samplingEffort	The amount of effort expended during an Event.
eventDate	The date-time or interval during which an Event occurred. For occurrences, this is the date-time when the event was recorded. Not suitable for a time in a geological context.
habitat	A category or description of the habitat in which the Event occurred.
eventRemarks	Comments or notes about the Event.
locationID	An identifier for the set of location information (data associated with dcterms:Location). May be a global unique identifier or an identifier specific to the dataset.
country	The name of the country or major administrative unit in which the Location occurs.
stateProvince	The name of the next smaller administrative region than country (state, province, canton, department, region etc.) in which the Location occurs.
county	The full, unabbreviated name of the next smaller administrative region than stateProvince (county, shire, department etc.) in which the Location occurs.
locality	The specific description of the place. Less specific geographic information can be provided in other geographic terms (higherGeography, continent, country, stateProvince, county, municipality, waterBody, island, islandGroup). This term may contain information modified from the original to correct perceived errors or standardise the description.
minimumElevationInMeters	The lower limit of the range of elevation (altitude, usually above sea level), in metres.
locationRemarks	Comments or notes about the Location.
verbatimCoordinates	The verbatim original spatial coordinates of the Location. The coordinate ellipsoid, geodeticDatum or full Spatial Reference System (SRS) for these coordinates should be stored in verbatimSRS and the coordinate system should be stored in verbatimCoordinateSystem.
decimalLatitude	The geographic latitude (in decimal degrees, using the spatial reference system given in geodeticDatum) of the geographic centre of a Location. Positive values are north of the Equator, negative values are south of it. Legal values lie between -90 and 90, inclusive.
decimalLongitude	The geographic longitude (in decimal degrees, using the spatial reference system given in geodeticDatum) of the geographic centre of a Location. Positive values are east of the Greenwich Meridian, negative values are west of it. Legal values lie between -180 and 180, inclusive.
geodeticDatum	The ellipsoid, geodetic datum or spatial reference system (SRS), upon which the geographic coordinates given in decimalLatitude and decimalLongitude are based.
coordinateUncertaintyInMeters	The horizontal distance (in metres) from the given decimalLatitude and decimalLongitude describing the smallest circle containing the whole of the Location. Leave the value empty if the uncertainty is unknown, cannot be estimated or is not applicable (because there are no coordinates). Zero is not a valid value for this term.
footprintWKT	A Well-Known Text (WKT) representation of the shape (footprint, geometry) that defines the Location. A Location may have both a point-radius representation (see decimalLatitude) and a footprint representation and they may differ from each other.
georeferencedBy	A list (concatenated and separated) of names of people, groups or organisations who determined the georeference (spatial representation) for the Location.
georeferenceRemarks	Notes or comments about the spatial description determination, explaining assumptions made in addition or opposition to the those formalised in the method referred to in georeferenceProtocol.

### Data set 2.

#### Data set name

occurrence.txt

#### Data format

Tab delimited Darwin Core Archive

#### Number of columns

30

#### Character set

UTF-8

#### 

**Data set 2.** 

Column label	Column description
occurrenceID	An identifier for the Occurrence (as opposed to a particular digital record of the occurrence). In the absence of a persistent global unique identifier, construct one from a combination of identifiers in the record that will most closely make the occurrenceID globally unique.
institutionID	An identifier for the institution having custody of the object(s) or information referred to in the record.
collectionID	An identifier for the collection or dataset from which the record was derived.
datasetID	An identifier for the set of data. May be a global unique identifier or an identifier specific to a collection or institution.
institutionCode	The name (or acronym) in use by the institution having custody of the object(s) or information referred to in the record.
collectionCode	The name, acronym, coden or initialism identifying the collection or dataset from which the record was derived.
basisOfRecord	The specific nature of the data record.
catalogNumber	An identifier (preferably unique) for the record within the data set or collection.
occurrenceRemarks	Comments or notes about the Occurrence.
recordNumber	An identifier given to the Occurrence at the time it was recorded. Often serves as a link between field notes and an Occurrence record, such as a specimen collector's number.
recordedBy	A list (concatenated and separated) of names of people, groups or organisations responsible for recording the original Occurrence. The primary collector or observer, especially one who applies a personal identifier (recordNumber), should be listed first.
individualCount	The number of individuals represented, present at the time of the Occurrence.
reproductiveCondition	The reproductive condition of the biological individual(s) represented in the Occurrence.
occurrenceStatus	A statement about the presence or absence of a Taxon at a Location.
associatedTaxa	A list (concatenated and separated) of identifiers or names of taxa and their associations with the Occurrence.
identifiedBy	A list (concatenated and separated) of names of people, groups or organisations who assigned the Taxon to the subject.
dateIdentified	The date on which the subject was identified as representing the Taxon.
identificationReferences	A list (concatenated and separated) of references (publication, global unique identifier, URI) used in the Identification.
identificationRemarks	Comments or notes about the Identification.
nameAccordingToID	An identifier for the source in which the specific taxon concept circumscription is defined or implied. See nameAccordingTo.
scientificName	The full scientific name, with authorship and date information, if known. When forming part of an Identification, this should be the name in lowest level taxonomic rank that can be determined. This term should not contain identification qualifications, which should, instead, be supplied in the IdentificationQualifier term.
kingdom	The full scientific name of the kingdom in which the taxon is classified.
phylum	The full scientific name of the phylum or division in which the taxon is classified.
class	The full scientific name of the class in which the taxon is classified.
order	The full scientific name of the order in which the taxon is classified.
family	The full scientific name of the family in which the taxon is classified.
genus	The full scientific name of the genus in which the taxon is classified.
specificEpithet	The name of the first or species epithet of the scientificName.
infraspecificEpithet	The name of the lowest or terminal infraspecific epithet of the scientificName, excluding any rank designation.
taxonRank	The taxonomic rank of the most specific name in the scientificName.

### Data set 3.

#### Data set name

multimedia.txt

#### Data format

Tab delimited Darwin Core Archive

#### Number of columns

11

#### Character set

UTF-8

#### 

**Data set 3.** 

Column label	Column description
identifier	An arbitrary code that is unique for the resource, with the resource being either a provider, collection or media item.
type	dc:type may take as value any type term from the DCMI Type Vocabulary. Recommended terms are Collection, StillImage, Sound, MovingImage, InteractiveResource, Text. Values may be used either in their literal form or with a full namespace from a controlled vocabulary, but the best practice is to use the literal form when using dc:type and use dcterms:type when you can supply the URI from a controlled vocabulary and implementers may require this practice. At least one of dc:type and dcterms:type must be supplied but, when feasible, supplying both may make the metadata more widely useful. The values of each should designate the same type, but in case of ambiguity dcterms:type prevails.
subtype	Any URI may be used that provides for more specialisation than the type. Possible values are community-defined. For examples, see the non-normative page AC_Subtype_Examples.
MetadataDate	Point in time recording when the last modification to metadata (not necessarily the media object itself) occurred. The date and time must comply with the World Wide Web Consortium (W3C) datetime practice, which requires that date and time representation correspond to ISO 8601:1998, but with year fields always comprising 4 digits. This makes datetime records compliant with 8601:2004. AC datetime values may also follow 8601:2004 for ranges by separating two IS0 8601 datetime fields by a solidus ("forward slash", '/'). See also the Wikipedia IS0 8601 entry for further explanation and examples.
metadataLanguage	URI from the ISO639-2 list of URIs for ISO 3-letter language codes. Note: At least one of ac:metadataLanguage and ac:metadataLanguageLiteral must be supplied but, when feasible, supplying both may make the metadata more widely useful.
metadataLanguageLiteral	Language of description and other metadata (but not necessarily of the image itself) represented as an ISO639-2 three letter language code. ISO639-1 two-letter codes are permitted but deprecated.
providerManagedID	A free-form identifier (a simple number, an alphanumeric code, a URL etc.) that is unique and meaningful primarily for the data provider.
WebStatement	A URL defining or further elaborating on the licence statement (e.g. a web page explaining the precise terms of use).
associatedSpecimenReference	A reference to a specimen associated with this resource.
accessURI	A URI that uniquely identifies a service that provides a representation of the underlying resource. If this resource can be acquired by an http request, its http URL should be given. If not, but it has some URI in another URI scheme, that may be given here.
format	A string describing the technical format of the resource (file format or physical medium).

### Data set 4.

#### Data set name

extendedmeasurementorfact.txt

#### Data format

Tab delimited Darwin Core Archive

#### Number of columns

10

#### Character set

UTF-8

#### 

**Data set 4.** 

Column label	Column description
measurementID	An identifier for the MeasurementOrFact (information pertaining to measurements, facts, characteristics or assertions). May be a global unique identifier or an identifier specific to the dataset.
measurementType	The nature of the measurement, fact, characteristic or assertion. Recommended best practice is to use a controlled vocabulary.
measurementTypeID	An identifier for the measurementType (global unique identifier, URI). The identifier should reference the measurementType in a vocabulary.
measurementValue	The value of the measurement, fact, characteristic or assertion.
measurementValueID	An identifier for facts stored in the column measurementValue (global unique identifier, URI). This identifier can reference a controlled vocabulary (e.g. for sampling instrument names, methodologies, life stages) or reference a methodology paper with a DOI. When the measurementValue refers to a value and not to a fact, the measurementvalueID has no meaning and should remain empty.
measurementUnit	The units associated with the measurementValue. Recommended best practice is to use the International System of Units (SI).
measurementDeterminedDate	The date on which the MeasurementOrFact was made. Recommended best practice is to use an encoding scheme, such as ISO 8601:2004(E).
measurementDeterminedBy	A list (concatenated and separated) of names of people, groups or organisations who determined the value of the MeasurementOrFact.
measurementMethod	A description of or reference to (publication, URI) the method or protocol used to determine the measurement, fact, characteristic or assertion.
measurementRemarks	Comments or notes accompanying the MeasurementOrFact.
